# Protein 2B of Coxsackievirus B3 Induces Autophagy Relying on Its Transmembrane Hydrophobic Sequences

**DOI:** 10.3390/v8050131

**Published:** 2016-05-12

**Authors:** Heng Wu, Xia Zhai, Yang Chen, Ruixue Wang, Lexun Lin, Sijia Chen, Tianying Wang, Xiaoyan Zhong, Xiaoyu Wu, Yan Wang, Fengmin Zhang, Wenran Zhao, Zhaohua Zhong

**Affiliations:** 1 Department of Microbiology and Wu Lien-Teh Institute, Harbin Medical University, 157 Baojian Road, Harbin 150081, China; wuheng1990@163.com (H.W.); zhai_xia_cool@126.com (X.Z.); cy_hmu@126.com (Y.C.); katekitekite@163.com (R.W.); linlexun@163.com (L.L.); chensj0802@163.com (S.C.); wangty0929@163.com (T.W.); littlerock712@163.com (X.Z.); wangyan@hrbmu.edu.cn (Y.W.); fengminzhang@ems.hrbmu.edu.cn (F.Z.); 2 Department of Cell Biology, Harbin Medical University, 157 Baojian Road, Harbin 150081, China; 3 Department of Cardiology, The First Hospital of Harbin Medical University, 23 Youzheng Street, Harbin 150001, China; xiaoyu_wu2006@163.com (X.W.); 4 Department of Reproductive Medicine, The First Hospital of Harbin Medical University, 23 Youzheng Street, Harbin 150001, China

**Keywords:** coxsackievirus B3, autophagy, protein 2B, transmembrane hydrophobic sequence

## Abstract

Coxsackievirus B (CVB) belongs to *Enterovirus* genus within the *Picornaviridae* family, and it is one of the most common causative pathogens of viral myocarditis in young adults. The pathogenesis of myocarditis caused by CVB has not been completely elucidated. In CVB infection, autophagy is manipulated to facilitate viral replication. Here we report that protein 2B, one of the non-structural proteins of CVB3, possesses autophagy-inducing capability. The autophagy-inducing motif of protein 2B was identified by the generation of truncated 2B and site-directed mutagenesis. The expression of 2B alone was sufficient to induce the formation of autophagosomes in HeLa cells, while truncated 2B containing the two hydrophobic regions of the protein also induced autophagy. In addition, we demonstrated that a single amino acid substitution (56V→A) in the stem loop in between the two hydrophobic regions of protein 2B abolished the formation of autophagosomes. Moreover, we found that 2B and truncated 2B with autophagy-inducting capability were co-localized with LC3-II. This study indicates that protein 2B relies on its transmembrane hydrophobic regions to induce the formation of autophagosomes, while 56 valine residue in the stem loop of protein 2B might exert critical structural influence on its two hydrophobic regions. These results may provide new insight for understanding the molecular mechanism of autophagy triggered by CVB infection.

## 1. Introduction

Viral myocarditis caused by coxsackievirus B (CVB) infection is one of the most common conditions that mainly affects children and young adults [1,2,3,4,5]. In severe cases, CVB infection may result in sudden death or viral persistency which eventually leads to dilated cardiomyopathy [6,7,8]. The pathogenesis of CVB infection is believed to be the consequence of cellular damage or death due to virus replication [1,6]. In addition, the inflammatory and immune response induced by CVB infection also plays an important role in viral myocarditis [9,10,11,12,13]. In spite of the extensive investigations, the pathogenesis of CVB infection has not been fully understood.

CVB belongs to *Enterovirus* genus of *Picornaviredae* [6]. It is single-stranded, positive-sense RNA (ssRNA) virus. The icosahedrial capsid of the virus is composed of four viral structural proteins, VP1, VP2, VP3, and VP4. Inside the capsid of the virion, there is an ssRNA which contains a single open-reading frame (ORF). Eleven peptides are translated from the genome of CVB, including four capsid proteins, two viral proteases (2A and 3C), one RNA-dependent RNA polymerase (3D), three proteins involved in viral RNA synthesis (2B, 2C, and 3AB), and a small polypeptide VPg that binds the 5′ untranslated region (UTR) of viral RNA [4,6,14,15]. Among these viral non-structural proteins, 2B has been demonstrated to contain hydrophobic domains that enable it to insert into the membrane of the host cell [16,17].

Protein 2B of CVB is a small integral membrane polypeptide with 99 amino acids in length [18]. It contains two hydrophobic regions connected by a short stem loop. One hydrophobic region has been predicted to form an amphipathic α-helix, while the other forms a complete hydrophobic helix. This helix-loop-helix motif of 2B is believed to be the basis for 2B to form transmembrane pore by homo-multimerization [17,19]. In CVB-infected cells, 2B is found to localize in the membrane derived from Golgi apparatus and ER [19,20]. The pore-forming feature of 2B in the membrane of these organelles resulted in the decreased Ca^2+^ store in ER and Golgi apparatus [20,21]. It has been found that the expression of 2B enabled cells to resist apoptosis induced by certain stimuli, and this anti-apoptotic property of 2B-expressing cells relied on the reduced Ca^2+^ store in ER and Golgi complex [21,22]. However, the role of protein 2B in autophagy has not yet been identified.

Autophagy is the physiological catabolic process in which cells degrade internalized pathogens or worn-out organelles by the formation of membrane-enclosed autophagosomes [23,24]. Abnormality in autophagy has been found to be involved in a variety of conditions such as cancer, neurodegenerative diseases, and viral infection [24,25,26,27,28]. It has been demonstrated that CVB replication was supported by the assembly of autophagosomes [29]. Our previous study also showed that autophagic response was induced in cardiac myocytes in the mice infected with CVB3 [30]. However, the molecular mechanism by which CVB manipulates autophagy is poorly understood. The present study found that the expression of 2B alone was sufficient to induce autophagy. The autophagy-inducing motif is located in the region 36aa-83aa of protein 2B, which covers its entire hydrophobic sequences. In addition, 2B mutant in 56 valine residue (V→A) failed to induce autophagy, indicating the key role of this particular amino acid residue, which is located in between the two helices of protein 2B, in the induction of autophagy.

## 2. Materials and Methods

### 2.1. Antibodies and Chemicals

Rabbit anti-enhanced green fluorescent protein (EGFP) polyclonal antibody, anti-rabbit horseradish peroxidase-conjugated secondary antibody, and anti-actin antibody were obtained from Cell signaling (Danvers, MA, USA). Rabbit anti-LC3 polyclonal antibody was obtained from Sigma-Aldrich (St. Louis, MO, USA). Rabbit anti-enterovirus VP1 antibody was obtained from Dako (Shanghai, China). Prime STAR HS DNA polymerase, T4 DNA ligase, *Hind* III and *Xba* restrictive enzymes were obtained from TaKaRa (Dalian, China).

### 2.2. Cell Culture

HeLa cells were maintained by the Department of Microbiology, Harbin Medical University, Harbin, China. Cells were cultured in DMEM medium (Invitrogen, Shanghai, China) supplemented with 10% fetal calf serum (FCS) (Biological Industries), 100 units of penicillin/mL, and 100 mg of streptomycin/mL. Cells were grown at 37 °C in a 5% CO_2_ incubator.

### 2.3. Virus Infection

CVB3 Wooddruff was amplified in HeLa cells. Cells were grown to 70%–80% confluency and transfected with pMKS1 (a kind gift form Prof. J. Lindsay Whitton, The Scripps Research Institute, San Diego, CA, USA), which contains the cDNA of the entire genome of CVB3. Virus titers were measured by plaque-forming unit (pfu). The titer of CVB3 used in this study was 1.8 × 10^8^ pfu/mL. The multiplicity of infection (MOI) of 10 was used in this study.

### 2.4. Plasmid Construction

pmCherry-C1 (Clontech) was a kind gift from Prof. Wenhui Li, Beijing life science Institute, Beijing, China. pmCherry-LC3 was constructed based on pmCherry-C1. pcDNA3.1(+) was kindly provided by Prof. Hong Ling, Department of Microbiology, Harbin Medical University, Harbin, China. Plasmids expressing the of EGFP-2B and EGFP-2B truncated were constructed based on pcDNA3.1(+). Plasmids expressing EGFP-2B mutants were constructed based on pEGFP-2B by site-directed mutagenesis and overlapping polymerase chain reaction (PCR). Sequence analysis of the constructs was performed by Genewiz (Beijing, China). PCR primers were obtained from TaKaRa.

### 2.5. Transfection

HeLa cells were transiently transfected with plasmids as described previously [31]. Briefly, cells were cultured in 12-well culture plate for 24 h to 60%–70% confluency. Exponentially growing cells were transfected with plasmid mixed with Lipofectmine 2000 (Invitrogen). The expression of fluorescence was observed with confocal microscope CellVoyager CV1000 (Yokogawa, Japan).

### 2.6. Western Blot

Cells were washed twice with cold phosphorate-buffered saline (PBS) and then lysed on ice for 20 min with the treatment of RIPA lysis buffer (Thermo, Shanghai, China) containing protease inhibitor cocktail and 1% phenylmethylsulfonyl fluoride (PMSF) (Beyotime, China). Cell lysates were centrifuged at 12,000 g for 10 min at 4 °C. The protein concentration of the cell lysate was determined by Bradford assay with Protein Assay Kit (Bio-Rad, Hercules, CA, USA). Proteins were separated by 15% SDS-PAGE and transferred to polyvinylidene difluoride (PVDF) membrane (Millipore, Billerica, Massachusetts, USA). The membrane was blocked for 1 h with 5% skimmed milk dissolved in 1× TBS containing 0.3% Tween 20. The membrane was incubated overnight with primary antibodies at 4 °C. After three washes with TBS containing 0.3% Tween 20 and 5% skimmed milk, the membrane was incubated with secondary antibody for 2 h at room temperature. Protein bands were visualized by enhanced chemiluminescence technique using SuperSignal West Pico chemiluminescent substrate (Thermo, USA).

## 3. Results

### 3.1. CVB3 Replication Induces Autophagy in HeLa Cells

**Figure 1:**
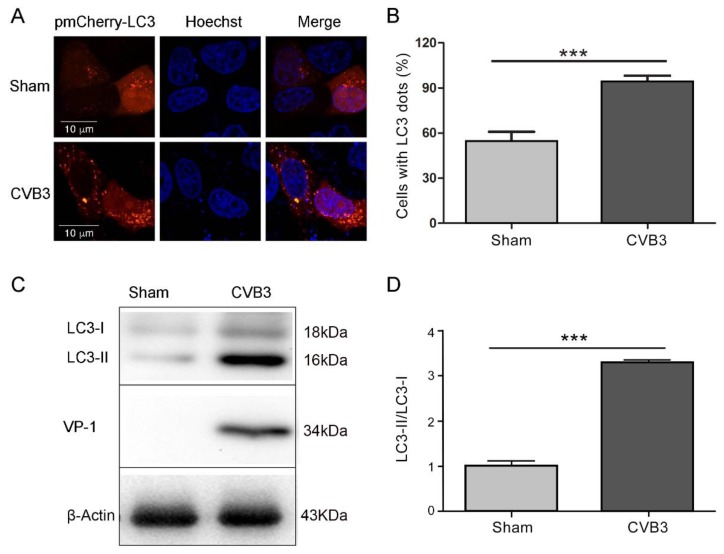
Coxsackievirus B 3 (CVB3) induces autophagy in HeLa cells. HeLa cells were infected with CVB3 (MOI = 10) for 7 h. Cells cultured in Dulbecco’s Modified Eagle Medium (DMEM) without CVB3 infection were taken as control. (**A**) Cells were observed by confocal microscope (×600); (**B**) cells with LC3 dots were counted over the LC3-expressing cells. *** *p* < 0.01. Data were derived from 100 cells in each sample; (**C**) VP1 and LC3 were detected by Western blotting; (**D**) the ratio of LC3-II/LC3-I was calculated according to the results of Western blots. *n* = 4. Experiment was repeated three times. Representative images were presented.

Our previous study has shown that CVB3 infection induces autophagy in HeLa cells and primary cardiomyocytes [30]. To show the autophagic response of HeLa cells triggered by CVB3 infection, HeLa cells were transfected with pmCherry-LC3 for 24 h and then were infected with CVB3 at MOI = 10 for 7 h. Cells were viewed by confocal microscope. The expression of CVB3 VP1 and LC3 was analyzed by Western blotting. As shown in Figure 1, CVB3 infection increased the number of cells with fluorescence pancta, while uninfected cells showed largely dispersed fluorescence distributed in the cytoplasm (Figure 1A,B). Western blot analysis showed that the level of LC3-II was dramatically increased in CVB3-infected cells compared with that in sham-infected cells (Figure 1 C,D). These data demonstrated that the replication of CVB3 induced autophagic response in HeLa cells.

### 3.2. Protein 2B of CVB3 Induces Autophagy

**Figure 2:**
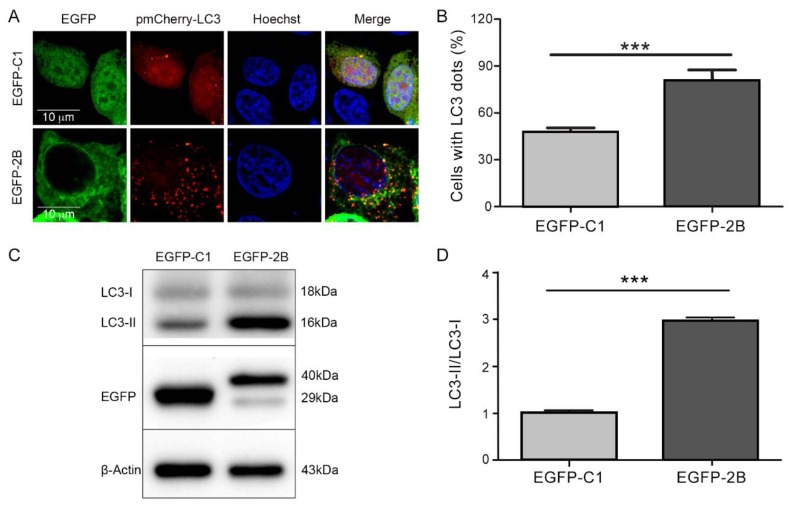
Protein 2B of CVB3 induces autophagy. (**A**) HeLa cells were co-transfected with pEGFP-2B and pmCherry-LC3 for 48 h. The control cells were co-transfected with pEGFP-C1 and pmCherry-LC3. Cells were observed by confocal microscope (×600); (**B**) cells with LC3 dots were counted over the LC3-expressing cells. *** *p* < 0.01. Data were derived from 100 cells in each sample; (**C**) HeLa cells were transfected with pEGFP-2B for 42 h and treated with E64-D/PEPA (10 ng/mL) for 6 h. Enhanced green fluorescent protein (EGFP) and LC3 were determined by Western blotting; (**D**) the ratio of LC3-II/LC3-I was calculated according to the results of Western blots. *n* = 4. Experiment was repeated four times. Representative images were presented.

Studies have shown that the non-structural protein 2B of CVB is an integral membrane protein, which disturbs the permeability of plasma membrane and cytoplasmic membrane. However, the role of protein 2B in autophagy has not been studied. To determine whether or not protein 2B could induce autophagy, HeLa cells were co-transfected with pEGFP-2B and pmCherry-LC3 for 48 h. The fluorescence distribution and the expression of LC3 were determined by confocal microscopy and Western blotting. As it is shown in Figure 2, cells transfected with pEGFP-2B showed punctate pattern of LC3 fluorescence distribution (Figure 2A,B) and increased level of LC3-II expression (Figure 2C,D). These findings indicate that the expression of 2B alone is sufficient to induce autophagy. Our results also showed that protein 2B was co-localized with LC3-II in autophagosomes (Figure 2A).

### 3.3. The Autophagy-Inducing Motif of Protein 2B

2B of CVB3 is an integral membrane protein. It contains two hydrophobic regions linked by a loop. The first hydrophobic region of protein 2B forms an amphipathic α-helix. These regions together form a helix-loop-helix motif, which is predicted to traverse cellular membrane and to assemble into ionic pore [19]. Autophagy is a process in which the assembly of autophagosmes surrounding the worn-out cellular structures and long-lived proteins predominates [24]. Since protein 2B is an integral membrane protein, we speculate that its transmembrane hydrophobic regions might be responsible for the induction of autophagy.

**Figure 3:**
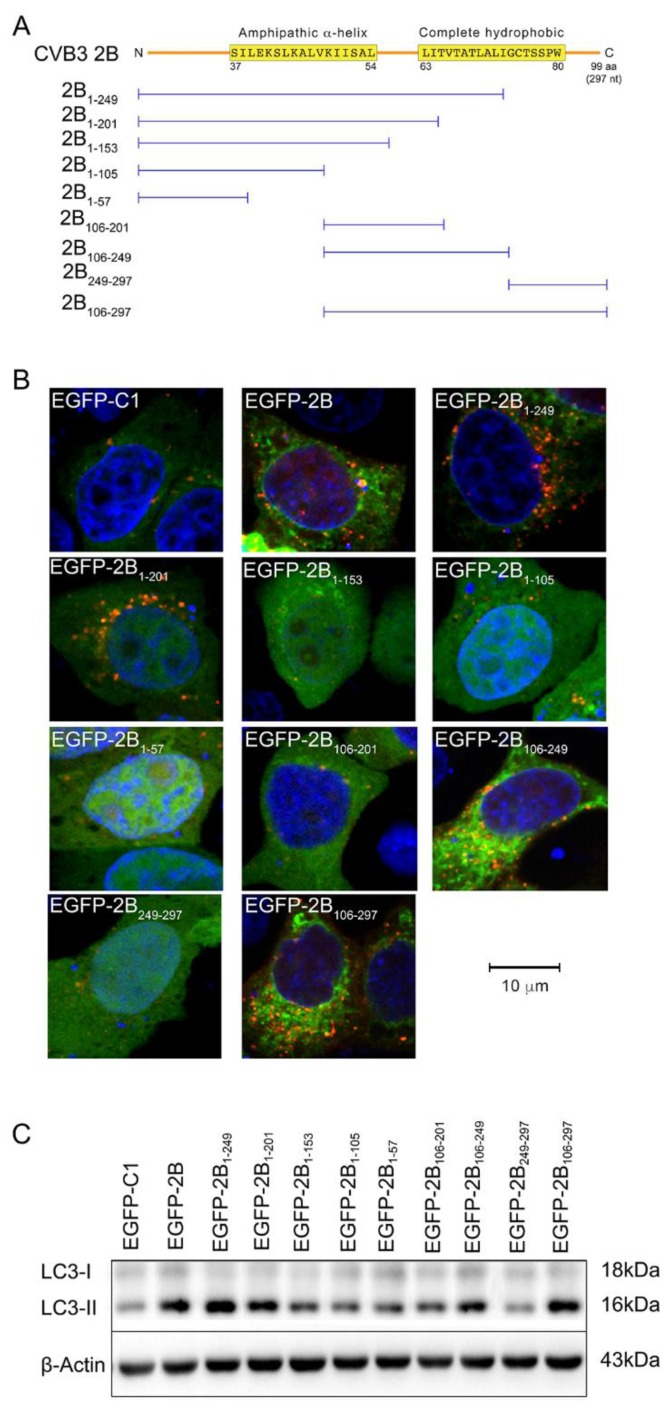
The autophagy-inducing motif of protein 2B. (**A**) The diagram shows the constructs expressing truncated protein 2B fused with EGFP (not included in the plot); (**B**) HeLa cells were co-transfected with pEGFP-2B_XX_ (representing the truncated 2B) and pmCherry-LC3 for 42 h and treated with E64-D/PEPA (10 ng/mL) for 6 h. The control cells were transfected with pEGFP-C1 and pmCherry-LC3. Cells were observed by confocal microscope (×600); (**C**) HeLa cells were treated as described in (**B**). EGFP and LC3 were detected by Western blotting. *n* = 4. Experiment was repeated three times. Representative images were presented.

To identify the autophagy-inducing sequences of protein 2B, plasmids expressing truncated 2B were constructed. These plasmids were designated as pEGFP-2B_1-249_, pEGFP-2B_1-201_, pEGFP-2B_1-153_, pEGFP-2B_1-105_, pEGFP-2B_1-57_, pEGFP-2B_106-201_, pEGFP-2B_106-249_, EGFP-2B_249-297_, and pEGFP-2B_106-297_, respectively, according to the fragments of 2B that they express (Figure 3A). HeLa cells were co-transfected with pmCherry-LC3 and the pEGFP-2B_XX_ (“_XX_” represents the truncated 2B). The expression of truncated 2B is shown in the Supplimentary materials (Figure S1). Like the effect of full-length 2B, punctate pattern of LC3 fluorescence was observed in the cells transfected with pEGFP-2B_1-249_, pEGFP-2B_106-249_, and pEGFP-2B_106-297_, respectively (Figure 3B). Cells transfected with these constructs also showed increased expression of LC3-II (Figure 3C). According to the amino acid sequence of CVB3 (GenBank: U57056.1), 2B_1-249_, 2B_106-249_, and 2B_106-297_ contain the helix-loop-helix motif. This finding indicates that the membrane spanning sequence and the loop region of 2B are required for the induction of autophagy. Our results also showed that 2B in full length, 2B_106-249_, and 2B_106-297_ co-localized with LC3-II in autophagosomes (Figure 3B).

As is also shown in Figure 3B, 2B_1-105_ and 2B_1-153_ failed to induce autophagy. These truncated 2B lack either the entire helix-loop-helix motif or part of the motif. This observation further demonstrates that the helix-loop-helix motif is essential for the induction of autophagy.

It is also worth noting that 2B_1-201_ and 2B_106-201_, both of which contain the amphipathic α-helix, the loop region, and the five initial amino acids (from 63L to 67T) in the second hydrophobic region of 2B, showed distinct capability in autophagy-induction. 2B_1-201_ induced the formation of autophagosomes, while 2B_106-201_ did not.

### 3.4. 56 Valine Residue in between the Transmembrane Sequences of Protein 2B is Essential for Autophagy-Induction

**Figure 4:**
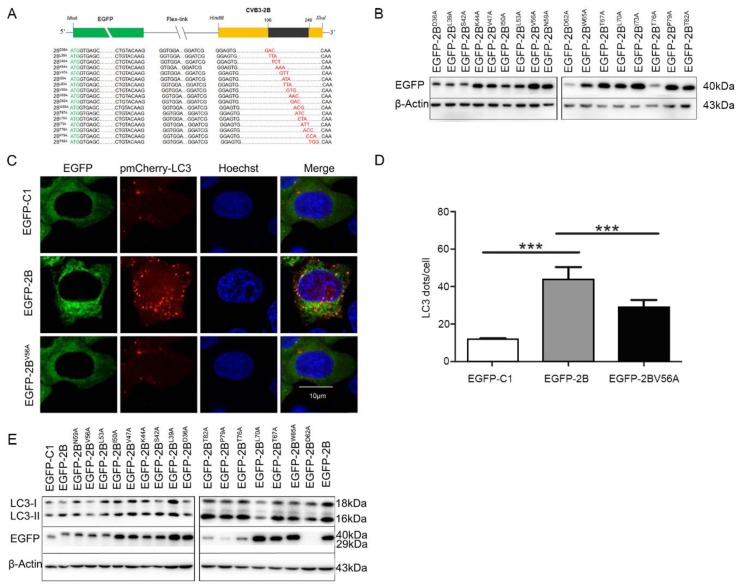
Mutated 2B^V56A^ failed to induce autophagy. (**A**,**B**) Plasmids expressing EGFP-mutated 2B with single amino acid substitution were generated by site-directed mutagenesis. The expression of 2B mutants were analyzed by Western blotting; (**C**) HeLa cells were co-transfected with pEGFP-2B^V56A^, which expresses the mutant 2B (56V→A) fused with EGFP, and pmCherry-LC3 for 42 h and treated with E64-D/PEPA at 10 ng/mL for 6 h. Control cells were co-transfected with pEGFP-C1 and pmCherry-LC3. Cells were observed by confocal microscope (×600); (**D**) the statistic results show LC3 puncta in each cell from the results of (**C**). *** *p* < 0.01. Data are derived from 15 cells for each sample; (**E**) HeLa cells were transfected with pEGFP-2B mutant for 42 h and treated with E64-D/PEPA at 10 ng/mL for 6 h. Control cells were transfected with pEGFP-C1. EGFP and LC3 were analyzed by Western blotting. *n* = 4. Experiment was repeated three times. Representative images were presented.

To further confirm the amino acid sequence of protein 2B that is responsible for the induction of autophagy, plasmids expressing mutated 2B with single substitution of amino acid were constructed by site-directed mutagenesis. Cells were co-transfected with the construct expressing the EGFP-mutated 2B and pmCherry-LC3. Confocal microscopy and Western blotting were used to determine the autophagic response. As shown in Figure 4C, no fluorescence puncta occurred in the cells transfected with pEGFP-2B^V56A^. Moreover, the level of LC3-II in the cells transfected with pEGFP-2B^V56A^ remained unchanged (Figure 4E). Fluorescence puncta were observed in the cells transfected with the plasmids expressing the other mutated 2B (data not shown). Correspondingly, the levels of LC3-II were also increased in various extent in the cells transfected with most of the plasmids expressing mutant 2B. Notably 2B^V56A^ showed no increase (Figure 4E). Taken together, these data indicate that the amino acid 56V in between the transmembrane sequences of protein 2B is indispensable for 2B-induced autophagy.

## 4. Discussion

Autophagy is a conserved catabolic process that is essential for cellular homeotasis. Evidence has shown that a variety of viruses could manipulate autophagy to assist their replication [24]. It has been demonstrated that CVB3 replication induced the formation of autophagosomes which may act as viral RNA replication sites [29,32]. Our previous study showed that autophagic response was induced by CVB3 infection in the cardiac myocytes of mice [30]. To further explore the molecular mechanism of autophagy-inducing property of CVB3, viral non-structural protein 2B was studied.

The non-structural proteins of CVB are essential participants in viral RNA replication, the assembly of virions, and the release of viruses. For instance, 2A and 3C protease cleave the polypeptide translated from viral genome, a single ORF, into viral structural and non-structural proteins [1]. Viral protease 2A and 3C have also been demonstrated to cleave a plethora of cellular proteins to facilitate viral replication or to inhibit innate immunity [33,34,35].

Protein 2B of *Picornaviridae* family has been reported to be involved in the reorganization of cytoplasmic membrane during virus replication [36]. However, the disturbing influence of protein 2B on cellular activities is far from clear.

2B is an integral membrane protein encoded by CVB. It contains two hydrophobic regions connected by a short loop. The first hydrophobic region (aa 37–54) forms an amphipathic α-helix, while the second complete hydrophobic region (aa 63–80) also forms a helix structure [19,20,37]. This helix-loop-helix motif is believed to be the foundation for 2B to form complex through homo-multimerization in cellular membrane [17]. Evidence has shown that protein 2B assembles into ionic pore in plasma membrane and cytoplasmic membrane [19,20]. The pore-formation property of 2B enables it to regulate intracellular Ca^2+^ concentration through promoting the influx of Ca^2+^ from extracellular matrix as well as the efflux of Ca^2+^ from ER to cytosol [21]. In addition, it has been demonstrated that the pore-forming feature of 2B facilitates CVB release from the host cell. Evidence also showed that the expression of 2B alone also endows the cell to resist apoptosis induced by certain stimuli. This anti-apoptosis property is related with the Ca^2+^-regulating feature of 2B [22]. However, whether or not protein 2B of CVB plays a role in autophagy is unknown.

Here in agreement with our previous study and the studies by others [29,30], CVB3 infection induced the formation of autophagosomes, which were represented by the fluorescence puncta of LC3 in the cytoplasm. More importantly, we found that the expression of 2B alone was sufficient to induce the formation of autophagosomes in which 2B and LC3 co-localized. To reveal the precise amino acid sequence which might be involved in the induction of autophagy, a series of truncated 2B peptides were constructed. Our results showed that cells expressing the peptides containing the complete helix-loop-helix motif induced autophagy. On the other hand, truncated 2B containing incomplete helix-loop-helix motif failed to induce autophagy. This finding suggests that the transmembrane property of protein 2B mediated by its two stretches of hydrophobic sequence is required for its autophagy-inducing function. Our results are supported by the report of de Jong in which both hydrophobic regions of protein 2B were found to be required for its proper membrane association [37].

Amino acid substitution study showed that the 56 valine residue, which resides in between the amphipathic α-helix and the second hydrophobic region, plays a critical role in 2B-induced autophagy. According the predicted architecture of protein 2B, 56V belongs to the loop region which links the two hydrophobic regions [17]. Although 56V contains non-polar side chain, it could be inferred that this particular amino acid residue or the loop region might exert critical influence on the two transmembrane regions of protein 2B. In consistence with our postulation, it has been reported that the hydrophilic region between the two helices is critical for the multimerization of 2B in Golgi complex [38]. Nonetheless, further study is needed.

This study also found that both protein 2B and the truncated 2B with autophagy-inducing capability were co-localized with LC3-II. Autophagy is a process which requires the assembly of membranous structures, the phagophores and autophagosomes [39]. Protein 2B is an integral membrane protein which is involved in the re-arrangement of cytoplasmic membrane [20,40]. Therefore, the involvement of 2B in the induction of autophagy might be a logical process. Since protein 2B is mainly located in Golgi complex and ER with efficient pore-forming property [37,41], our finding implies that Golgi complex and ER might be the major membrane source for the assembly of autophagosomes in 2B-expressing cells and likely in CVB3-infected cells. However, further study is needed to reveal the molecular mechanism involved.

We also noted that two truncated 2B, 2B_1-201_ and 2B_106-201_, showed distinct autophagy-inducing feature. Both fragments above contain the first helix and the loop region of 2B, while the C-terminal region ahead of the α-helix is lacking in 2B_106-201_. These results seem to indicate the importance for the C-terminal sequence of protein 2B in autophagy-induction. However, truncated 2B containing only the C-terminal sequence did not induce autophagy. It should be noted that, although truncated 2B constructs provide a direct way to identify specific amino acid sequences involved in autophagy, these 2B fragments certainly do not represent the real situation during CVB infection.

Taken together, here we found that protein 2B of CVB3 could induce autophagy. The autophagy-inducing motif resides in the transmembrane hydrophobic regions of protein 2B, while 56V in the loop region of 2B is critical for the induction of autophagy. This study may provide new insight for understanding the mechanism of autophagy induced by CVB3 infection and to develop antiviral compounds by targeting protein 2B.

## Supplementary Materials

The following material is available online. Table S1: Sequences of PCR primers for site-directed mutagenesis of 2B. Figure S1: The expression of truncated 2B in HeLa cells.

## Abbreviations

The following abbreviations are used in this manuscript:

CVB	coxsackievirus B
UTR	untranslated region
ORF	open reading frame
MOI	multiplicity of infection
pfu	plaque-forming unit
EGFP	enhanced green fluorescence protein
LC3	microtubule-associated protein 1light chain 3
PCR	polymerase chain reaction
