# Supplementary material for: Protein 2B of Coxsackievirus B3 Induces Autophagy Relying on Its Transmembrane Hydrophobic Sequences
Source: Viruses. 2016 May 12;8(5):131. doi: 10.3390/v8050131 (PMC4885086; doi:10.3390/v8050131)
Supplement: Supplementary file 1 [file viruses-08-00131-s001.zip › viruses-119870-supplementary-revised-proofread/Legend of Figure S1.docx]

**Figure S1**: The expression of the truncated 2B in HeLa cells. HeLa cells were transfected with pEGFP-2Bxx (“xx” represents truncated 2B) for 48 h. Cells were observed with fluorescence microscope (×400). Cell nuclei were stained with DAPI.
