# Supplementary material for: Protein 2B of Coxsackievirus B3 Induces Autophagy Relying on Its Transmembrane Hydrophobic Sequences
Source: Viruses. 2016 May 12;8(5):131. doi: 10.3390/v8050131 (PMC4885086; doi:10.3390/v8050131)
Supplement: Supplementary file 1 [file viruses-08-00131-s001.zip › viruses-119870-supplementary-revised-proofread/Supplementary material-Table S1.docx]

**Table S1. Sequences of PCR primers for the site-directed mutagenesis of 2B**

| Primer name | | Primer sequence |
| --- | --- | --- |
| 2B^pro^ | PF | ATATAT***AAGCTT***GGAGTGAAGGACTATGTGGAAC |
| 2B^pro^ | PR | ATAGCG***TCTAGA***TTATTGGCGTTCAGCCATGGGTA |
| 2B^D36A^ | PF | AGTGGGTCAAGCCTCCATCTTAGA |
| 2B^D36A^ | PR | TCTAAGATGGAGGCTTGACCCACT |
| 2B^L39A^ | PF | CAAGACTCCATCGCAGAGAAGTCTCTAAAAG |
| 2B^L39A^ | PR | CTTTTAGAGACTTCTCTGCGATGGAGTCTTG |
| 2B^S42A^ | PF | CCATCTTAGAGAAGGCTCTAAAAGCCTTAGTT |
| 2B^S42A^ | PR | AACTAAGGCTTTTAGAGCCTTCTCTAAGATGG |
| 2B^K44A^ | PF | GAGAAGTCTCTAGCGGCCTTAGTTAA |
| 2B^K44A^ | PR | TTAACTAAGGCCGCTAGAGACTTCTC |
| 2B^V47A^ | PF | CTAAAAGCCTTAGCCAAGATAATATCAGCC |
| 2B^V47A^ | PR | GGCTGATATTATCTTGGCTAAGGCTTTTAGAG |
| 2B^I50A^ | PF | GTTAAGATAGCCTCAGCCTTAGTAATTGTG |
| 2B^I50A^ | PR | CACAATTACTAAGGCTGAGGCTATCTTAAC |
| 2B^L53A^ | PF | AGATAATATCAGCCGCAGTAATTGTGGTG |
| 2B^L53A^ | PR | CACCACAATTACTGCGGCTGATATTATCT |
| 2B^V56A^ | PF | GCCTTAGTAATTGCCGTGAGGAACC |
| 2B^V56A^ | PR | GGTTCCTCACGGCAATTACTAAGGC |
| 2B^N59A^ | PF | TTGTGGTGAGGGCCCACGATGACCTAAT |
| 2B^N59A^ | PR | ATTAGGTCATCGTGGGCCCTCACCACAA |
| 2B^D62A^ | PF | AACCACGATGCACTAATCACGGTGACTG |
| 2B^D62A^ | PR | CAGTCACCGTGATTAGTGCATCGTGGTT |
| 2B^W65A^ | PF | CGATGACCTAATCGCGGTGACTGCCACACT |
| 2B^W65A^ | PR | AGTGTGGCAGTCACCGCGATTAGGTCAT |
| 2B^T67A^ | PF | AATCACGGTGGCTGCCACACTAG |
| 2B^T67A^ | PR | GCTAGTGTGGCAGCCACCGTGATT |
| 2B^L70A^ | PF | TGACTGCCACAGCAGCCCTCATTGGTTG |
| 2B^L70A^ | PR | CAACCAATGAGGGCTGCTGTGGCAGTC |
| 2B^I73A^ | PF | ACACTAGCCCTCGCTGGTTGTACCTCGT |
| 2B^I73A^ | PR | ACGAGGTACAACCAGCGAGGGCTAGTG |
| 2B^T76A^ | PF | ATTGGTTGTGCCTCGTCCCCATGGCGGT |
| 2B^T76A^ | PR | ACCGCCATGGGGACGAGGCACAACCAAT |
| 2B^P79A^ | PF | TTGTACCTCGTCCGCATGGCGGTGGCTT |
| 2B^P79A^ | PR | AAGCCACCGCCATGCGGACGAGGTACAA |
| 2B^T82A^ | PF | CCATGGCGGGCGCTTAAGCAGAAAGTGT |
| 2B^T82A^ | PR | ACACTTTCTGCTTAAGCGCCCGCCATGG |

Bold ltalic: The endonuclease recognition site for *Hind* III (AAGCTT) and *Xba* I (TCTAGA).

Underlined: the mutated site in the sequence of 2B.
