# Supplementary figures and images for: Protein 2B of Coxsackievirus B3 Induces Autophagy Relying on Its Transmembrane Hydrophobic Sequences
Source: Viruses. 2016 May 12;8(5):131. doi: 10.3390/v8050131 (PMC4885086; doi:10.3390/v8050131)

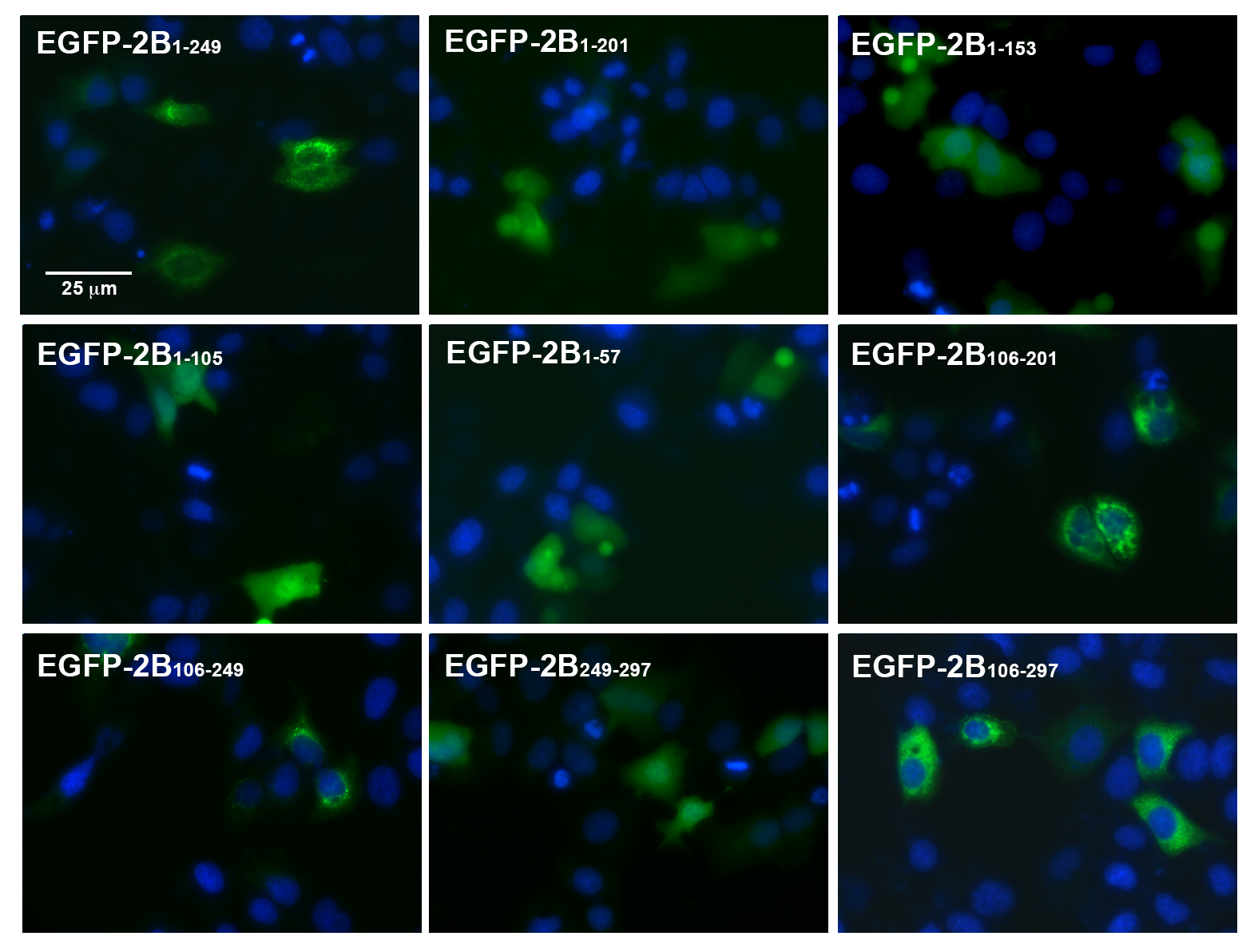

Supplement: Supplementary file 1 [file viruses-08-00131-s001.zip › viruses-119870-supplementary-revised-proofread/Figure S1.tif]
